# Unveiling the hidden economic toll of biological invasions in the European Union

**DOI:** 10.1186/s12302-023-00750-3

**Published:** 2023-06-08

**Authors:** Morgane Henry, Brian Leung, Ross N. Cuthbert, Thomas W. Bodey, Danish A. Ahmed, Elena Angulo, Paride Balzani, Elizabeta Briski, Franck Courchamp, Philip E. Hulme, Antonín Kouba, Melina Kourantidou, Chunlong Liu, Rafael L. Macêdo, Francisco J. Oficialdegui, David Renault, Ismael Soto, Ali Serhan Tarkan, Anna J. Turbelin, Corey J. A. Bradshaw, Phillip J. Haubrock

**Affiliations:** 1 Department of Biology, McGill University, Montréal, QC Canada; 2 Institute for Global Food Security, School of Biological Sciences, Queen’s University Belfast, Belfast, BT9 5DL UK; 3 School of Biological Sciences, King’s College, University of Aberdeen, Aberdeen, AB24 3FX UK; 4 Center for Applied Mathematics and Bioinformatics, Department of Mathematics and Natural Sciences, Gulf University for Science and Technology, Hawally, Kuwait; 5 Estación Biológica de Doñana, CSIC, Avda. Americo Vespucio 26, 41092 Seville, Spain; 6 Faculty of Fisheries and Protection of Waters, University of South Bohemia in České Budějovice, South Bohemian Research Centre of Aquaculture and Biodiversity of Hydrocenoses, Zátiší 728/II, 389 25 Vodňany, Czech Republic; 7 GEOMAR Helmholtz-Zentrum für Ozeanforschung Kiel, Düsternbrooker Weg 20, 24105 Kiel, Germany; 8 Université Paris-Saclay, CNRS, AgroParisTech, Ecologie Systématique Evolution, Gif sur Yvette, France; 9 Bioprotection Aotearoa, Lincoln University, Lincoln Canterbury, 7647 New Zealand; 10 Department of Sociology, Environmental and Business Economics, University of Southern Denmark, Degnevej 14, 6705 Esbjerg Ø, Denmark; 11 UMR 6308, AMURE, Université de Bretagne Occidentale, IUEM, rue Dumont d’Urville, 29280 Plouzané, France; 12 Marine Policy Center, Woods Hole Oceanographic Institution, Woods Hole, MA 02543 USA; 13 College of Fisheries, Ocean University of China, Qingdao, 266003 China; 14 Institute of Hydrobiology, Chinese Academy of Sciences, Wuhan, 430072 China; 15 Graduate Program in Conservation and Ecotourism, Federal University of Rio de Janeiro State, Rio de Janeiro, RJ Brazil; 16 Neotropical Limnology Group (NEL), Federal University of Rio de Janeiro State, Av. Pasteur, 458, Rio de Janeiro, RJ 22290-240 Brazil; 17 University of Rennes, CNRS, ECOBIO (Ecosystèmes, Biodiversité, Evolution), UMR, 6553 Rennes, France; 18 Institut Universitaire de France, 1 rue Descartes, 75231 Paris Cedex 05, France; 19 Department of Basic Sciences, Faculty of Fisheries, Muğla Sıtkı Koçman University, 48000 Muğla, Turkey; 20 Department of Life and Environmental Sciences, Faculty of Science and Technology, Bournemouth University, Poole, Dorset UK; 21 Global Ecology | Partuyarta Ngadluku Wardli Kuu, College of Science and Engineering, Flinders University, Adelaide, SA 5001 Australia; 22 ARC Centre of Excellence for Australian Biodiversity and Heritage (EpicAustralia.org.au), Wollongong, NSW Australia; 23 Department of River Ecology and Conservation, Senckenberg Research Institute and Natural History Museum Frankfurt, Gelnhausen, Germany

**Keywords:** Projection, InvaCost, Monetary impacts, Invasion costs, Temporal trends, Missing data

## Abstract

**Background:**

Biological invasions threaten the functioning of ecosystems, biodiversity, and human well-being by degrading ecosystem services and eliciting massive economic costs. The European Union has historically been a hub for cultural development and global trade, and thus, has extensive opportunities for the introduction and spread of alien species. While reported costs of biological invasions to some member states have been recently assessed, ongoing knowledge gaps in taxonomic and spatio-temporal data suggest that these costs were considerably underestimated.

**Results:**

We used the latest available cost data in *InvaCost* (v4.1)—the most comprehensive database on the costs of biological invasions—to assess the magnitude of this underestimation within the European Union via projections of current and future invasion costs. We used macroeconomic scaling and temporal modelling approaches to project available cost information over gaps in taxa, space, and time, thereby producing a more complete estimate for the European Union economy. We identified that only 259 out of 13,331 (~ 1%) known invasive alien species have reported costs in the European Union. Using a conservative subset of highly reliable, observed, country-level cost entries from 49 species (totalling US$4.7 billion; 2017 value), combined with the establishment data of alien species within European Union member states, we projected unreported cost data for all member states.

**Conclusions:**

Our corrected estimate of observed costs was potentially 501% higher (US$28.0 billion) than currently recorded. Using future projections of current estimates, we also identified a substantial increase in costs and costly species (US$148.2 billion) by 2040. We urge that cost reporting be improved to clarify the economic impacts of greatest concern, concomitant with coordinated international action to prevent and mitigate the impacts of invasive alien species in the European Union and globally.

**Supplementary Information:**

The online version contains supplementary material available at 10.1186/s12302-023-00750-3.

## Background

Invasive alien species—those introduced into regions where they are not native and in which they cause negative impacts to nature and/or society—are among the main drivers of global biodiversity decline [1, 2], and considered a growing threat with multiple feedbacks to ecosystem services and human well-being [3–6]. The ecological and socio-cultural impacts of invasive alien species are substantial and expected to continue increasing due to climate [7], geopolitical [8], and economic changes [9].

Most assessments of the impacts of invasive alien species have relied on scoring systems, such as those proposed by Panetta [10] and subsequently updated by Kumschick et al. [11] and Vilà et al. [12]. However, scoring systems are often context-dependent, with some scoring criteria lacking objectivity or requiring advanced assessor expertise [13], rendering them unsuitable for broader generalisation or prediction. There is therefore a need to improve the reliability of impact assessments by considering monetary costs arising from the loss and damage of resources as well as management actions. Attempts to quantify the economic costs of invasive alien species at broad spatial scales have occurred in recent decades [14], although early attempts had considerable shortcomings [15] or were limited to economic inventories of specific sectors [16, 17].

Most recently, the *InvaCost* project and the structured and publicly available database it has produced [18–20], have provided an unprecedented opportunity to investigate taxonomic and geographic trends in the monetary costs of invasive alien species worldwide (e.g., [21, 22]. Studies with a national (e.g., [23]), regional (e.g., [24]), continental (e.g., [25]), taxonomic (e.g., [26]), or pathway (e.g., [27, 28]) focus have, however, all identified difficulties with making comprehensive monetary cost estimates owing to spatial and taxonomic gaps. These data gaps include: (*i*) costs for many known, established invasive alien species that have not been assessed; (*ii*) cost information that is often inaccessible or otherwise not available publicly, or (*iii*) many cost estimates that do not fulfil quality criteria (i.e., peer-reviewed and/or transparent calculations).

In Europe, the monetary costs incurred by invasive alien species from 1960 to 2020 were recently estimated to be US$140.2 billion [22], although persistent data gaps suggest that this is an underestimate [29]. This inferred cost might appear low relative to the annual gross domestic product of some European countries (e.g., 3.3% when compared to Germany's 2021 gross domestic product [GDP] of US$4.3 trillion; data.worldbank.org). However, it is a more substantial quantity when considering countries with smaller economic turnover such as Hungary (US$182.3 billion) or Slovakia (US$115.0 billion).

The combined economic turnover of European Union member states makes it the third largest economy worldwide (data.worldbank.org), and Europe as a whole has been a historic centre of trade, human migration, and tourism. As such, both continental Europe and the European Union (with its current 27 member states) have been particularly vulnerable to biological invasions, despite recent transboundary legislation to tackle invasive alien species [30–33]. The European Union Invasive Alien Species Regulation 1143/2014 [34] directs efforts to tackle the threats presented by invasive alien species to the European Union. However, many such species, including the 88 identified species of Union concern (of which 12 are thought not to be present yet in the Union’s territory), lack any evidence of economic costs [19], despite the European Union being an economic and monetary union with coordinated economic and fiscal policies (e.g., a common monetary policy and currency). The problem is compounded because there are already several thousand established alien species in the European Union [35], with large discrepancies between the total number of established invasive alien species and those with assessed costs (for instance, France has a conservative minimum of 2621 alien species, but only 98 species [< 4%] with reported costs; [23]).

An absence of information on economic impacts risks downplaying the threats posed by invasive alien species (including costs and other impacts), because it underestimates the economic threat biological invasions represent and hinders national policies, governance, research, and educational efforts along with broad-scale multinational initiatives to combat the problem [36–38]. Improved quantification of these species' impacts is also essential for prioritisation, mitigation, and eradication that underlie the Convention on Biological Diversity 2020 [39], the 2022 Kunming-Montreal Global Biodiversity Framework, and the European Union Invasive Alien Species Regulation 1143/2014 [34]. However, even when implemented, such actions are most often locally focused, and are frequently of insufficient length or magnitude to address the scale of the problems because of budget limitations. This lack of allocated funding compromises outcomes while simultaneously increasing both long-term management and damage costs [40, 41]. Thus, the feasibility of management remains impaired by the political choice not to ensure adequate resources [42].

Because cost information for many invasive alien species is missing, national and transnational estimates would benefit from a more complete accounting. Given that invasion history is one of the best predictors of eventual impacts [43], using existing cost estimates of a measured species would seem the most appropriate and straightforward approach to interpolate missing costs in other invaded countries. However, context is also important, and we would not necessarily expect impacts to be identical across countries [5]. For instance, macroeconomic differences could influence the cost of labour (and management) or the per-unit currency value of a sector, and hence, the magnitude of damages. Alternatively, wealthier or larger countries could be better prepared to respond to invasions, resulting in inverse relationships between costs and macroeconomic indicators [44]. Given the challenges triggered by a lack of detailed cost information, we projected the available data to fill both spatial and taxonomic gaps to assess the extent to which we have so far underestimated the costs of invasive alien species to the economies of European Union member states. We also forecast how baseline invasion costs and factors correlated with costs (i.e., the number of costs reported in the literature and number of species with reported costs) will develop up to 2040 using several temporal modelling approaches. As a result, we develop a more comprehensive accounting of the costs of alien species to the European Union using invasion history and fitted macroeconomic scaling factors.

Our analyses are therefore important methodological and applied advances towards improving spatial and temporal estimates of costs. We expected that (*i*) the projected amount of costs of invasive alien species will be considerably higher than those reported in the *InvaCost* (v4.1) database due to (*ii*) mismatches between known invaders and those with recorded costs [23, 45–47], and (*iii*) expected increasing future trajectories of costs of invasions [19, 48, 49]. Our study makes a critical step towards improving cost predictions of biological invasions for the European Union as well as elsewhere and represents a substantial development beyond existing representations of their monetary impacts.

## Methods

### Cost data

To estimate the cost of biological invasions on the combined economy of the European Union, we used the latest available version of the *InvaCost* database (v4.1; [19]). *InvaCost* currently includes 13,553 entries of reported economic costs from invasive alien species retrieved from peer-reviewed, official, or grey-literature sources in both English and 21 other languages [45], and over 60 descriptor variables (i.e., impacted sector, type of cost, etc.). *InvaCost* (v4.1) has standardised individual cost records to a common currency and year to account for variation and inflation: 2017 US$ (see [19] for detailed information on conversion; exchange rate for 2017: US$1 = €0.8852; World Bank, 2022). We applied the conversion (except for the temporal projection; see below) for the period following 1960, because we could not obtain monetary exchange rates from official institutions (e.g., World Bank) prior to that year. We then converted costs from 2017 US$ to 2022 EU€ values using an inflation correction factor = 1.10545941 for the period between 2017 and 2022 and an average currency exchange rate for 2022 of US$1 = €0.9515 (World Bank, 2022).

To ensure that we used cost data from only the 27 member states, we selected costs for every individual country currently in the European Union (excluding candidate countries), using the 'Official_country' column from the *InvaCost* database: Austria, Belgium, Bulgaria, Croatia, Cyprus, Czech Republic, Denmark, Estonia, Finland, France (excluding French overseas territories), Germany, Greece, Hungary, Ireland, Italy, Latvia, Lithuania, Luxembourg, Malta, Netherlands, Poland, Portugal, Romania, Slovakia, Slovenia, Spain, and Sweden. This filtering step identified 5,442 cost entries from the original global dataset (13,553 entries).

To obtain comparable costs of invasive alien species, we considered all costs lasting for a period of < 1 year as 'annual costs', and re-calculated (and thus ‘expanded’) costs covering multiple years on an annual basis by spreading the total cost across the time covered, using the *expandYearlyCosts* function in the invacost package version 0.3–4 [20] in R version 4.1.3 [50]. Deriving the total cumulative cost of invasions over time requires taking into account the probable duration of each cost occurrence. The duration is the number of years between the probable starting ('Probable_starting_year') and ending ('Probable_ending_year') years of the costs reported by each publication included in the *InvaCost* database [19].

Expansion resulted in 7,214 annualised, European Union-relevant cost entries. To exclude cost estimates with doubtful reliability, we first filtered the *InvaCost* database to obtain a 'highly reliable, observed' dataset of only 'observed' costs using the 'Method_reliability', the ‘Method_reliability_refined’ and 'Implementation' columns reflecting (*i*) the perceived reliability of the type of publication and/or cost-estimation approach ('high', when originating from peer-reviewed articles, official reports, or from grey material but with documented, reproducible and traceable methods, and 'low' otherwise), and (*ii*) whether the cost was realised or empirically incurred ('observed') or whether it was based on predictions or costs expected over time or space ('potential'). The resulting dataset of 'highly reliable' and 'observed' costs contained 5,592 entries. Further analyses focused on two descriptors: (*i*) *type of cost*: 'damage' refers to damage or losses incurred by invasion, and 'management' includes control-related expenditures (i.e., costs due to monitoring, prevention, management, eradication); (*ii*) *impacted sector*: the activity, societal, or market sector incurring the cost (see Additional file 1).

### Taxonomic gaps

To identify taxonomic gaps in the reporting of potentially costly invasive alien species, we (*i*) quantified the ratio between recorded invasive alien species in *InvaCost* and established alien species nationally within European Union member states, and (*ii*) assessed the number of species of Union concern (last update, Commission Implementing Regulation (EU) 2022/1203 of 12 July 2022 Amending Implementing Regulation (EU) 2016/1141 to Update the List of Invasive Alien Species of Union Concern. Available online: https://eur-lex.europa.eu/legal-content/EN/TXT/HTML/?uri=CELEX:32022R1203&from=EN) on invasive alien species recorded in *InvaCost*. For this, we assembled the most comprehensive dataset of established alien species in the European Union by combining the *SInAS* database of alien species occurrences [51] with Casties et al. [52], as well by adding species that were not yet included in either of the two lists. We used the *SInAS_AlienSpeciesDB_2.4.1* [51] file as the base file for our dataset. We removed species without assignment of invaded country/region, or those not reported in the European Union from the dataset. Then, we also removed species with assignment only as CASUAL and ABSENT in the columns 'degreeOfEstablishment' (N) and 'occurrenceStatus' (L), respectively, due to their unclear establishment status in those regions [53]. Finally, we checked species identity and the spelling of scientific names against the Global Biodiversity Information Facility [54]. If we did not find a species in the Global Biodiversity Information Facility, we did general internet searches in June and July 2022 to confirm species authenticity. We corrected misspelt species names and removed any duplicate species from the dataset.

We applied a paired *t*-test to compare the total number of recorded, established alien species and those of Union concern listed within the dataset of established alien species in the European Union (github.com/LeungEcoLab/EU_costs), with the respective total number of invasive alien species recorded in *InvaCost* at the level of individual European Union member states. We also removed species not authenticated and thus, included only species-specific entries from the *InvaCost* database, thereby excluding any mixed or unspecified data.

### Spatial and sectoral projections

We used invasion history to interpolate costs to countries where a species was confirmed present using the dataset of established alien species in the European Union, but where the cost was not estimated, and also tested for differences due to macroeconomic factors. We restricted our interpolations only to species and sectors (e.g., agriculture, health, management and control, etc.) where a given species’ impact had been estimated in at least one European Union member state, and we only used highly reliable observed, country-level costs reported in the *InvaCost* database. We focused our analyses on country-level costs because they constitute most (92%) costs and are directly comparable. For full transparency, we also reported interpolated costs at the site level (see Additional file 1), although these are more difficult to compare because spatial scales of analyses varied widely, and no area information was available. Thus, our interpretation of interpolated costs at the site-level is speculative. After filtering for only highly reliable, observed costs, we obtained a total of 162 entries at the country level encompassing monetary costs of 49 species from 22 European Union member states. The site-level costs represented 2907 entries in 12 countries of the European Union for 179 species.

We also tested for differences resulting from macroeconomic factors and to account for potential socio-economic differences, we analysed the following: GDP, population size, country area, and importance of the impacted sector (in %). We expected costs to increase with GDP and population size because these are typical macroeconomic predictors with broad data availability, and they were relevant socio-economic predictors for the costs of invasive alien species in previous studies [22, 24, 55]. Further, the size (value) of a given sector logically relates to the potential magnitude of effect [56]. This last predictor scaled health and agriculture pest costs by the current health expenditure and the value added by agriculture, forestry, and fishery (both in % of GDP) (data.worldbank.org), respectively.

We scaled costs by the relative magnitudes of socio-economic factors, but costs need not change proportionally with macroeconomic factors, and a model should be flexible to account for this. For example, if the value of agriculture in country A was lower than in country B, the impact of an agricultural pest in A should also be fractionally, but not necessarily proportionally, lower than in B. We modelled relative differences in economic factors as a ratio (e.g., GDP_A_/GDP_B_), and accounted for non-proportional effects using a fitted coefficient $(\gamma$). In notation:

$$\hat{X}_{{s, c_{i} , h, j}} = \frac{1}{n}\mathop \sum \limits_{k = 1}^{n} X_{{s, c_{k} , h, j}} \mathop \prod \limits_{m = 1}^{M} \left( {\frac{{V_{{m, c_{i} }} }}{{V_{{m, c_{k} }} }}} \right)^{{\gamma_{m} }} ,$$

where $X$ is the *InvaCost-*listed cost, $\widehat{X}$ is the interpolated cost based on averaged, scaled observed costs, *s* is species, *c* is country, *h* is type of cost (damage, management, or both), and *j* is impacted sector. $V$ denotes each of the *M* macroeconomic variables analysed. *c*_*i*_ refers to each country *i* where species *s* has been observed, where we are trying to interpolate, whereas *c*_*k*_ refers to the country where species *k* has been both observed and measured in *InvaCost*. To avoid unreasonable extrapolations beyond the data, we truncated scalar ratios by the maximum (or minimum) observed in the fitted model. The model allows for effects ranging from proportional scaling of costs with macroeconomics ($\gamma$ = 1) to no effect ($\gamma$ = 0), and to either positive ($\gamma$> 0) or negative ($\gamma$< 0) effects.

We fitted values of $\gamma$ through maximum likelihood (using the *Optim* function in R), using the deviation between predicted costs (Eq. 1) and costs observed in *InvaCost* for species–sector combinations where there were entries across multiple countries. We considered alternative models using all possible combinations of our four macroeconomic variables and calculated the Akaike's information criterion (AIC) for each model (Additional file 1). Finally, we used the AIC-weighted average across all our models [57] to estimate the cost of each species–sector–country combination. We applied these values to species present in countries (Briski et al., unpublished data) but that were missing economic estimates. We estimated uncertainty in the parameter values $(\gamma )$ as well as the resulting economic estimates through bootstrapping.

### Temporal projection

To project the temporal dynamics of the monetary impacts at the European Union-level, we used the highly reliable and observed data subset. We then identified ‘extreme’ cost entries that can distort statistical analyses and violate model assumptions. These were any cost value exceeding the third quartile + 1.5 times the interquartile range for each year [21]. Using the *summarizeCosts* function implemented in the invacost package, we estimated the average cost for each decade in the time range 1970–2020 to visfit the raw cost trends over time because the first highly reliable, observed cost entry occurred in 1970. Accordingly, we did this analysis with the highly reliable, observed dataset with (*n* = 5592 entries) and then without (*n* = 4872 entries) ‘extreme’ cost entries.

To identify periods in which the variance of costs reported within *InvaCost* (v4.1) changed over time, we applied a first-order autoregressive AR (1) process for the residuals [58]. This enabled the computation of derivatives of fitted splines using the method of finite differences to estimate the rate of change (slope) in the fitted smoother [59]. This produces diagnostic plots of the costs over time, where we could identify periods of non-random change(s) and superimpose them on the respective temporal trend [60]. Following the non-random changes identified throughout the entire period, we identified a non-random change in costs after 1980 at which point a change in the trend is apparent and the few costs repeated over time prior to this point limited our ability to estimate the variance in costs. We therefore restricted this analysis to the period 1980–2017, excluding (*i*) the values reported from 2018 to 2020 due to lag times in reporting of costs after they are incurred [61, 62], and (*ii*) all years prior to 1980. We then modelled the annual total of this subset over time (using the 'Impact_year' of each cost entry as the year of occurrence) using both linear and quadratic robust regressions on the highly reliable, observed data via the *lmrob* function of the R robustbase package [63], with maximum iterations set to *n* = 1,000. Ultimately, we projected the trend in total costs until 2040, by using the *predict* function in the forecast R package [64]. Because we were interested in determining the projected trend of management expenditure in the European Union, we repeated the procedure considering only management costs—which constituted 90% of the entries with, and 93% of the entries without extreme values—by filtering the data using the 'Type_of_cost_merged' column. We therefore estimated both 'total' and 'management' costs separately and applied this analysis to our highly reliable observed data set and another subset without the identified extreme values (resulting in a final dataset of 3,685 cost entries). We did all analyses in R version 4.1.3 [50].

## Results

### Trends in documented costs

**Fig. 1:**
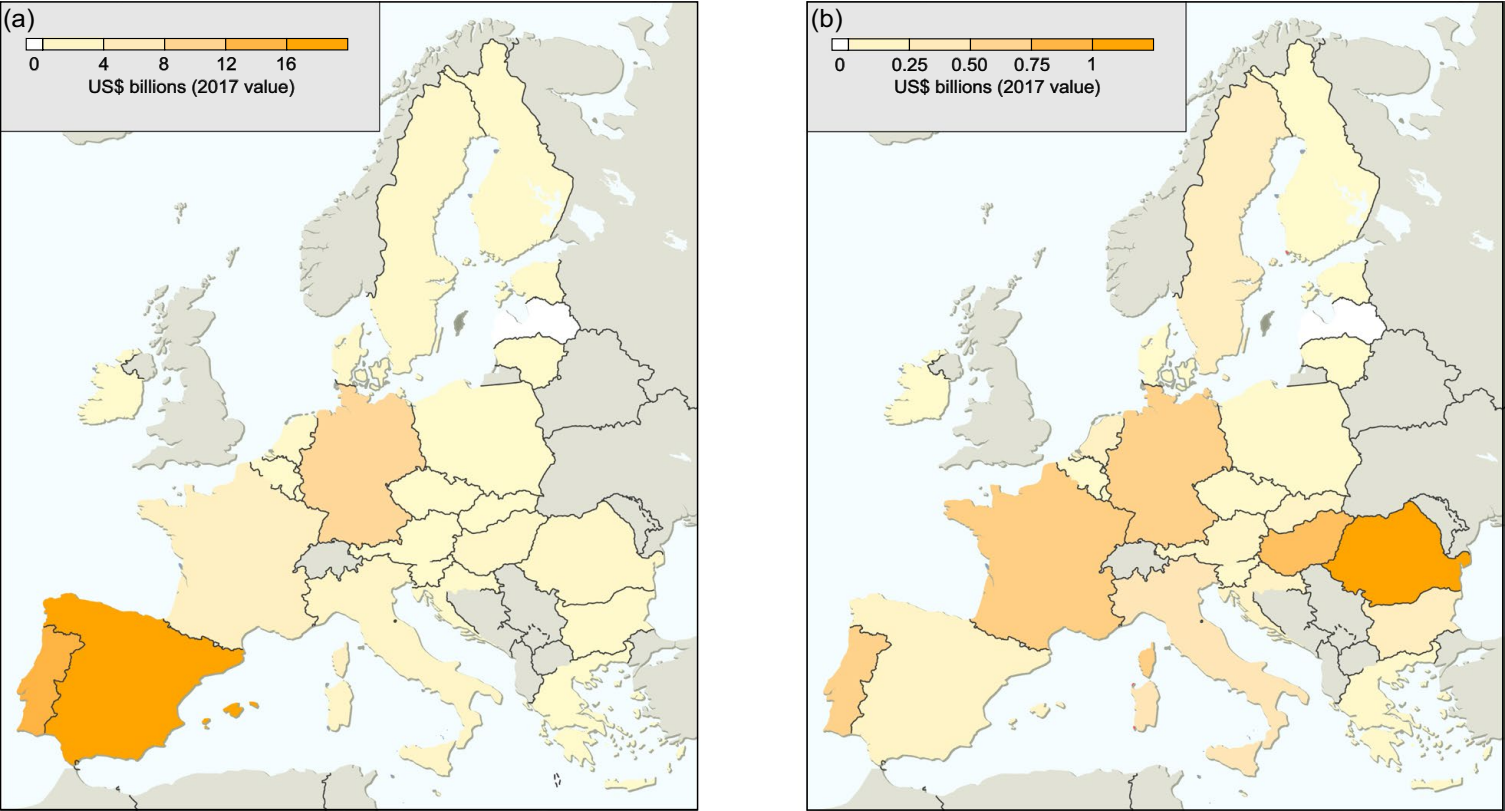
**a** Total invasion costs across European Union member states. **b** Highly reliable, observed cost subset across European Union member states. Note the different scales

The total cost of biological invasions across all European Union member states from 1960 to 2020 reported in *InvaCost* v4.1 amounted to US$129.9 billion (2017 value), corresponding to €138.6 billion (2022 value). Of these, US$7.3 billion (€7.7 billion; 5.9%) was highly reliable and observed, with US$752.9 million (€791.9 million; 10.28%) dedicated to management costs. Both total and highly reliable, observed costs varied substantially across European Union member states (Fig. 1; Additional file 1).

**Fig. 2:**
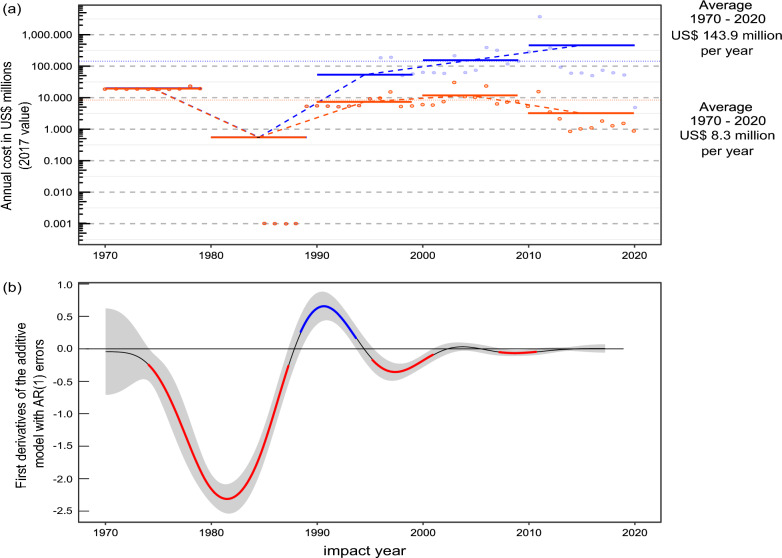
**a** Temporal trend of highly reliable, observed average annual costs per decade (solid bars) with (blue) and without (orange) extreme values recorded in *InvaCost* for European Union member states (annual costs are presented on the log_10_ scale). Points are annual totals, and the dash-dot line is the overall annual average. **b** Diagnostic plots showing changes in the total annual cost (highly reliable, observed subset) over time (considered against impact year that refers to the year that the cost incurred). Red: periods of non-random declines. Blue: periods of non-random increases

Including extreme values, the average annual cost from 1970 to 2020 was US$143.9 million (€151.4 million) although this varied substantially by decade, falling from an initial US$19.17 million (€20.17 million) annually in the 1970s to US$0.5 million in the 1980s, before increasing to US$53.3 million (€56.1 million) in the 1990s and ultimately reaching US$461.1 million (€485.0 million) annually in the 2010s (Fig. 2a). Excluding extreme values (Fig. 2a), the average annual cost from 1970 to 2020 was US$8.3 million (€8.7 million) and followed a similar decadal trend overall, increasing to US$11.6 million (€12.2 million) annually in the 2000s before a decrease in the 2010s. Decadal trends followed the projected change in reported costs over time, indicating the strongest increase between the 1980s and 1990s (Fig. 2b).

### Taxonomic gaps

**Fig. 3:**
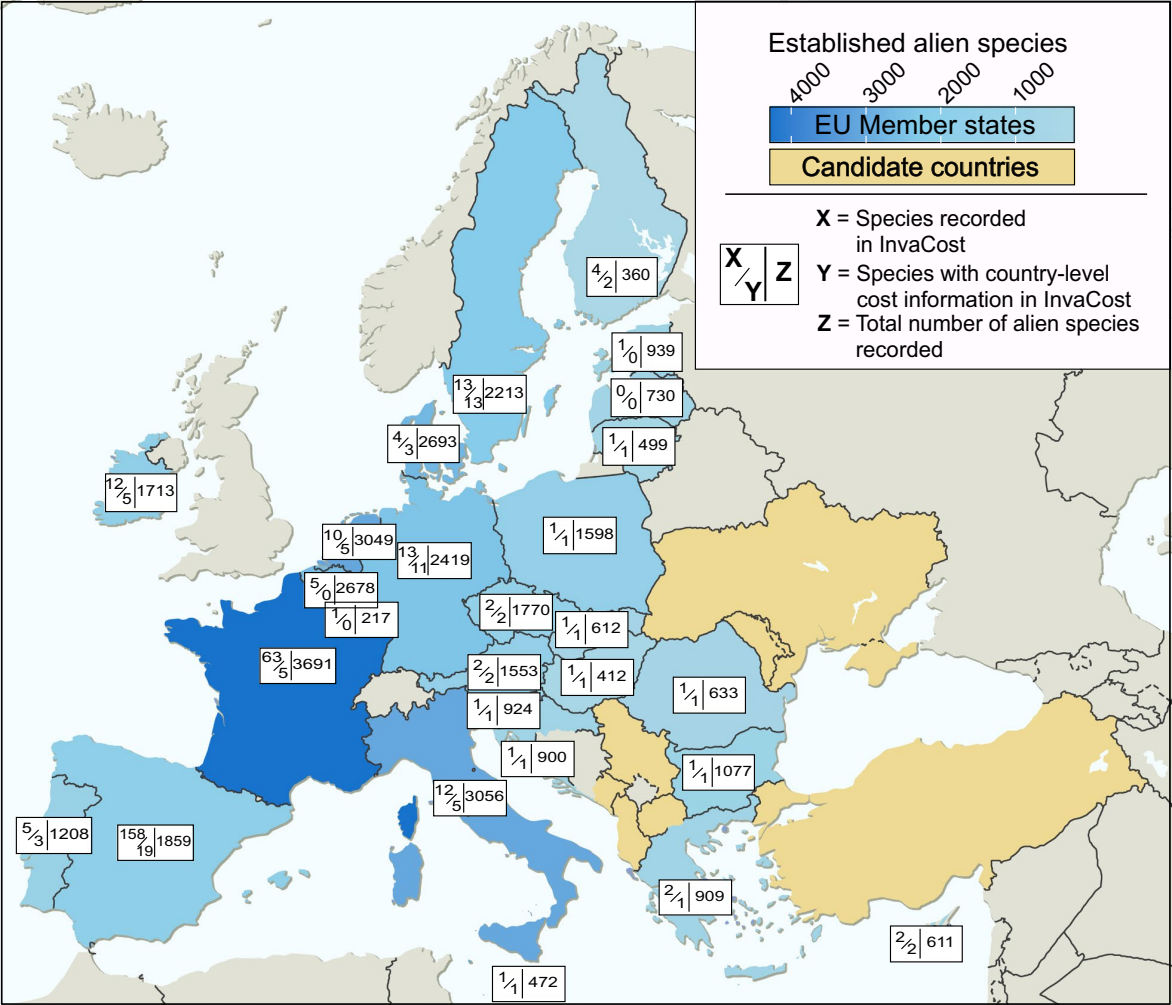
Contrasting information between invasive alien species with highly reliable, observed costs (*X*, box; upper left) and the subset of these that was reported at the country-level (*Y*, box; lower left) in *InvaCost* v4.1 and the total number of known alien species reported in Briski et al., unpublished data (Z, box; right) in each European Union member state (blue)

We identified 1459 (± 1011) established alien species on average recorded per European Union member state, ranging between 217 alien species established in Luxembourg and 3691 in France (Fig. 3; Additional file 1). In contrast, there was only an average of 15 (± 32) costly invasive alien species per European Union member state within *InvaCost* v4.1, ranging between one species in Luxembourg to 161 in Spain. This disparity implies that only ~ 1% of established alien species reported from European Union member states have recorded costs. Of the 88 species of Union concern, 66 are established in the European Union (Additional file 1), with 48 having reported economic cost in *InvaCost* (Additional file 1). On average, 21 (± 12) species of Union concern occur per European Union member state, but only 4 (± 8) have reported costs within that country. The number of established alien species by European Union member states recorded in our data and those listed as of Union concern were both statistically different from the member states’ respective number of invasive alien species in *InvaCost* (Briski et al.: *t*_26_ = 7.3786, *p* < 0.001; European Union Regulation 1143/2014: *t*_45_ = 6.0722, *p* < 0.001; Fig. 3).

### Spatial and sectoral taxonomic projections

Highly reliable, observed, species-specific costs totalled at US$6.5 billion (€6.8 billion), from which country-level costs (from 49 species; *n* = 162 entries) totalled US$4.7 billion (€4.9 billion; representing 89.8% of all highly reliable, observed costs entries for European Union member states). On this conservative basis of highly reliable, country-level costs, the different projections (Additional file 1) estimated the missing cost data to be ~ 869 cost entries across all European Union member states, contributing an additional US$23.4 billion (€24.6 billion) with uncertainties around the estimated missing costs after model averaging of a minimum 8.39 billion and maximum 54.9 billion, summing to a total of US$28.0 billion (€30.9 billion; + 501%). However, when excluding two high-leverage points from the final dataset (reported annualised costs of > $500 million as reported damage from Romania and Hungary), the averaged models (Additional file 1) estimated the missing costs for European Union member states at US$7.2 billion (€7.6 billion) with uncertainties around the estimated missing costs after model averaging of a minimum 6.43 billion and maximum 20.26 billion. Adding the missing costs resulted in a total of US$11.8 billion (€12.4 billion; + 153%) in this scenario. For a breakdown of cost imputations excluding high-leverage points, see Additional file 1.

**Fig. 4:**
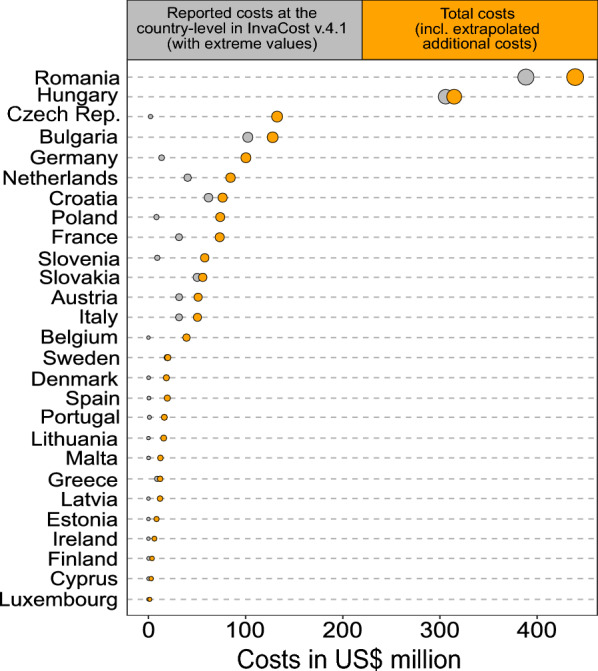
Comparison of highly reliable observed country-level costs and projected costs at the level of individual European Union member states. The size of the circles is scaled to the respective cost

The extent of projected costs across European Union member states varied substantially. The final dataset revealed that the highest increase in projected costs occurred in Lithuania (US$201.19 million; 1,093,525%), followed by Malta (US$193.25 million; 129,923%), Czech Republic (US$ 3,818.83 million; 60,093%), and Denmark (US$524.67 million; 54,670%). In terms of raw costs, the highest increase was in Czech Republic (US$3.8 billion; €4.0 billion), followed by Germany (US$2.4 billion; €2.5 billion), France (US$2.3 billion; €2.4 billion), the Netherlands and Poland both with US$2.0 billion (€2.1 billion). Added costs for all other countries were < US$2 billion (€2.1 billion). We provide a detailed breakdown of projected country-level costs in Fig. 4 and Additional file 1. On average, the projection of costs increased national invasion costs by 4750% (± 15,216%).

We also found that the projection of costs in European Union member states increased by an average of 2340% for impacted sectors (Additional file 1). Following the projection, costs to public and social welfare increased the most (US$4,243.49 million; 7950%), followed by costs to forestry (US$8,157.39; 4588%), authorities and stakeholders (US$9,333,86 million; 2611%), fisheries (US$88.28 million; 2112%), health (US$273.23 million; 30%), and the agricultural sector (US$549.73 million; 18%) (see Additional file 1 for a detailed description of the sectors considered in the *InvaCost* database). Finally, scenarios considering the missing costs of the two main types of costs resulted in projections increasing management costs by US$8,786.76 million (3933%), and by US$14,400.38 million (325%) for damage costs (Additional file 1).

### Temporal projection

**Fig. 5:**
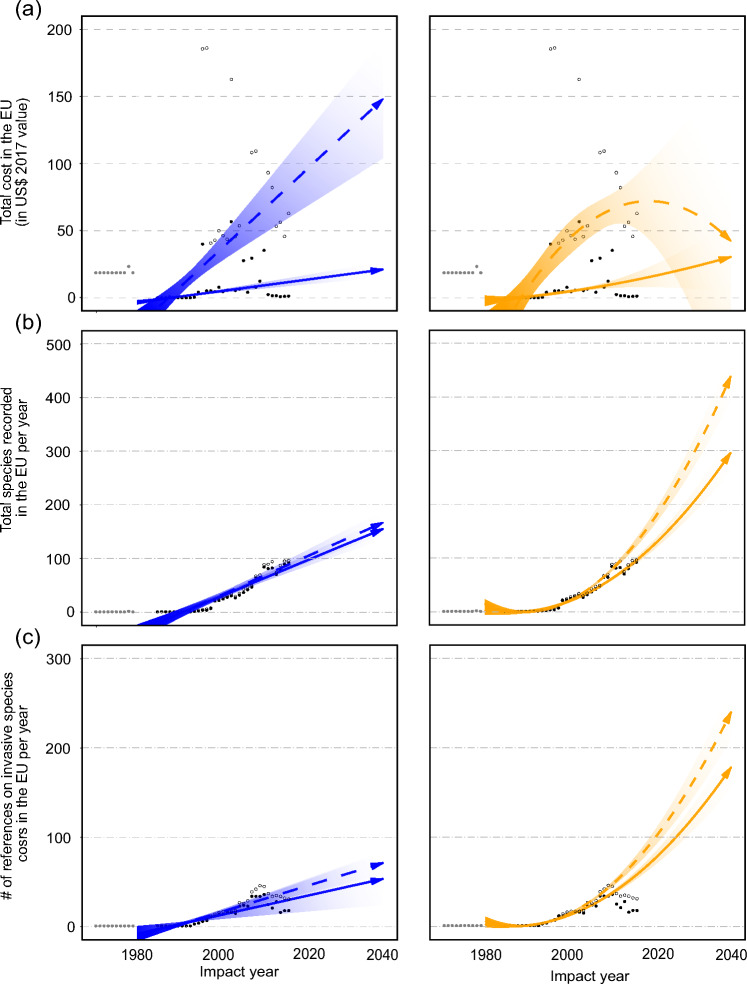
Temporal projection of total annual invasion costs (**a**), recorded species per year with costs (**b**), and number of reported references in *InvaCost* v4.1 per year (**c**) using linear (left, in blue) and quadratic (right, in orange) robust regressions with their respective confidence intervals. Solid dots and lines represent trends without the inclusion of extreme values. Open circles and dashed lines represent the respective trends when including all data (i.e., including extreme values). Grey dots present data for 1960–1979 that we did not consider in the projection due to an absence of repeated measures of individual cost entries

Using the 1980–2017 subset of highly reliable (observed costs), we identified substantial increases with both linear and quadratic robust regressions. This increase held even without extreme values, albeit reducing the US$7.3 billion (€7.7 billion) in highly reliable, observed costs to US$420.6 million (€442.4 million), with US$116.6 million (€122.7 million; 27.7%) associated with management expenditure. Linear and quadratic robust regressions estimated similar slopes, but including extreme values estimated a larger increase in projected trends. Projected costs for 2040 ranged from US$21.1 billion (€22.2 billion) to US$30.6 billion (€32.2 billion) excluding extreme values; and from US$42.2 billion (€44.4 billion) to US$148.2 billion (€155.9 billion) including extreme values. This was also reflected in projected increases in the total number of invasive alien species (155–296 annually without extreme values; 166–439 with extreme values; Fig. 5) and the number of references reporting costs (53–178 annually without extreme values; 71–240 with extreme values). Projected management costs followed similar trajectories but at lower magnitudes to those of overall costs for all measures (Additional file 1).

## Discussion

Data gaps in taxonomic, economic sectors, and country-level cost caused by biological invasions within the European Union are pervasive. Notwithstanding a conservative total cost of US$7.3 billion (€7.7 billion) that has already been incurred, we have shown that after accounting for taxonomic and geographic gaps in cost reporting, the magnitude of invasion costs was at least 1.5 to 6 times higher, even while using a conservative subset of the available data. We expect these costs to rise further towards 2040 in accordance with the temporal increase in numbers of alien species [48]. In addition, we found that the presence of high-impact (i.e., highly costly) species increased decadal averages by a factor of 10, underlining the importance of understanding the impacts and effective management actions for these particularly costly invasive alien species.

### Spatial, sectoral, and taxonomic projections

Economic costs are available for just 1% of alien species recorded as established across the European Union. For the 88 invasive alien species currently identified as being of Union concern, a much higher proportion of 54.5% had reported costs (although many still lacked costs evaluated at the level of individual member states). This absence of data is concerning given that ~ 14,000 alien taxa are listed in the *European Alien Species Database* [65] for Europe, and that invader distribution assessments can be incomplete or decades out of date [66]. This disparity between species with cost records and invader richness assessments indicates that the full economic burden on the European Union will be much higher than currently reported. Indeed, it is precisely this low proportion of alien species with reported costs at national levels that is one of the major limitations to estimating true total costs and raising awareness of the severe impacts of invasions across the European Union.

This underestimation is further underlined for multiple, highly impactful taxa that either have extensive potential distributions under current and future scenarios of environmental change [67, 68], or are present and have the potential to spread from the 32 neighbouring countries into the European Union. Further, we did not include in our analysis the European Union candidate countries (Albania, Moldova, Republic of North Macedonia, Montenegro, Serbia, Turkey, Ukraine), nor other European countries beyond the European Union, such as Switzerland, the United Kingdom, and Norway. When accounting for some of these gaps, cost projections revealed a potentially > six-fold (>500%) rise using only a conservative subset including only country-level entries from 49 species, thereby excluding 172 species (*n* = 3080 entries) with costs inferred only at the level of specific sites. We found that growth due to the scaling of missing costs was particularly burdensome to the public and social-welfare sector, followed by sectors encompassed by the circular economy initiatives and the European Green Deal (i.e., fisheries and forestry, both economic sectors of high importance to the European Union; [69, 70].

Although the projected cost increased by only 164% when we excluded two high-leverage points, we emphasise that this model was a poorer fit than the model including all values. While this does not necessarily mean that the higher projection is the most realistic, it does underline the importance of assessing the robustness of outliers. Here, these two high-leverage costs were both highly reliable and observed and thus not unreliable per se, as exemplified by massive costs arising from national eradication programmes of single species [71] or a few ‘hyper-costly’ invasive alien species [55].

Our cost modelling has taken into account all management costs, regardless of whether they were effective or inefficient, and hence we did not differentiate between, e.g., costly eradication measures and cheaper mitigation measures (i.e., based on efficacy or effectiveness). This information is often unavailable in underlying studies, but would constitute a major step forward in understanding management decisions for future work [72]. However, while costs from eradication efforts can be substantial, these can be short-term expenses for long-term savings [73, 74], and inevitably increase with per-capita effort over time as the populations or individuals most difficult to eradicate are targeted last, leading to an unequal temporal distribution of management costs. There is the possibility that such a substantial increase in projected management costs could undermine enthusiasm for future management efforts. However, it is also likely that when such control and eradication (a component of management costs) are successful, future costs are reduced to routine monitoring while avoiding the costs of inaction [61, 75].

Although not all alien species do or will cause monetisable damage or elicit management expenditure (e.g., non-monetised negative environmental impacts or incompatibility with current economic sectors; [76, 77]), the identified disparity with invader richness indicates that the full economic burden on the European Union will be much higher than the estimate we provide here. This reinforces the need for a coordinated effort across the European Union to adopt better reporting of invasion costs, and for central and accessible collation, as found within *InvaCost*, a living database that has grown considerably in only a few years (v0, 2017: 260 entries; v1.0: 2,419; v3.0: 9,823; v4.1, 2022: 13,553). Simplifying the process of adding information to such databases would facilitate use and uptake. This should include increasing outreach to industry groups, health insurance sectors, and others where a lack of communication among those who are affected by damage or loss, those who assess costs, and those who synthesise costs results in currently recorded but inaccessible data. Such efforts would provide a more complete and accurate picture of the total costs and the full economic impacts of biological invasions. While technical updates to database accessibility are complex and ongoing, our scaled projection approach provides a more complete estimate of costs and a major advance in the interpolation of invasion costs from limited data. Unlike previous attempts to investigate the temporal dynamics of the damage costs of invasive alien species [61, 75], the scaling model we developed permits spatial cost projections from one region to another given regional-specific species records or context-specific differences in conditions (e.g., economic factors). Such extrapolated costs then provide a basis for modelling incurred costs across a range of spatio-temporal scales.

### Temporal projection

Early cost reporting for invasive alien species was particularly sporadic (e.g., reporting high cost from the rabbit *Oryctolagus cuniculus* in Germany in the 1970s; [78]), with countries joining the European Economic Community in the 1980s and the first international research programmes (ERASMUS) being founded, before the foundation of the European Union in 1993. Hence, our models revealed further increases in costs, new costly invasive alien species, but also cost reports across the European Union over the next 20 years until 2040. Variation in decadal costs remained low, likely due to the conservative approach to include costs used to inform these temporal projections and exclude costs characterised as 'potential', meaning that they had not materialised at the time of reporting. However, it is possible that some of those costs could have been incurred following the year of reporting and that they might not have yet been recorded in *InvaCost*. The projected increase in the number of invasive alien species reflects a robust predicted rise identified in invasion rates and an increasing awareness of their impacts [79].

The well-characterised effect of reporting lags in the costs of invasive alien species [22, 20, 80] most likely explains the declines in total annual costs reported in recent years. An increasing number of alien species [7, 79] enabled by changing global climates and transport networks [81] will demand increasing management interventions and thereby inflate their future monetary burden, even though not all of them will become invasive [82]. This is particularly emphasised by reported management costs contributing 90–93% of the available data, while contributing only ~ 11% of the highly reliable, observed costs and ~ 28% when we excluded extreme values, and showing the steepest projected incline (Additional file 1). This increasing burden could also be exacerbated by continued economic growth, given the positive relationship between costs and economic output [22], changes in the economic output of sectors over time where resident invasive alien species impose the most damage, as well as reduced efficacy of management (e.g., via chemical resistance).

### Uncertainties

While projecting unknown costs of biological invasions in the European Union, our research has revealed large disparities between the total number of invasive alien species that have established themselves and those that have had their associated costs assessed (1%). Out of all the European Union member states, Romania and Hungary have notably higher reported costs resulting from invasive species at the country level. These high costs are driven by estimated damages caused by the common ragweed *Ambrosia artemisiifolia* and can potentially be explained by the large proportion of agricultural land invaded (36% and 61% in Romania and Hungary, respectively; [83]). This indicates how single species can have a disproportionate effect on cost reporting depending on the prevalence of impacted sectors within a location or region.

Because we estimated potential costs caused by invasive species for European Union member states where monetary impacts were unknown, it is important to emphasise the inherent uncertainty involved. Consequently, projected costs, whether over space or time, should be considered with caution. Nevertheless, we also note that these estimations were based only on a conservative subset of the richest country-level data, while considering that for invasive alien species from which models were fit, the costs were still fragmented and underestimated, thereby highlighting the importance of the costs involved. An additional source of uncertainty lies in lack of knowledge about the efficiency of management costs. Information on whether management expenditure has turned out to be efficient and to what degree, in relation to their objectives, can be essential for building a more comprehensive understanding of the true costs associated with biological invasions.

Our analyses do not consider any positive effects of invasive alien species, which are generally not captured in the *InvaCost* database. Examples could include, for instance, an increase in biological production in semi-arid ecosystems, the restoration of industrial or contaminated areas where native species cannot survive, with benefits from the acceleration of succession and soil formation or reductions in nutrient leakage and water basin eutrophication [84, 85], or fast-growing plants that increase carbon sequestration while outcompeting native species [86]. Alternatively, some invasive alien species such as *Buddleja davidii* might provide certain local economic benefits [87, 88], although these cannot discount or undermine the presence of costs because they often affect different actors and ecosystem functions [89], and nor have they been documented or known to be anywhere close to the magnitude of costs.

## Outlook

The number of invasive alien species and resultant economic costs will likely increase in the future, even if projections remain uncertain ﻿[7, 90], due to reporting lags [20], the emergence of new invasive alien species as costly [9], the high variance in reported costs between 1980 and 2020, and the low sample of cost estimates before 1980 [91]. Even so, the reported total of US$7.3 billion in highly reliable observed costs is a substantial sum, especially considering that fewer than 1% of alien species within the European Union have documented costs. Our results strongly indicate the need for collective mitigation and prevention actions within the European Union to prevent biological invasions and their associated costs. These include, in addition to existing legislation: (*i*) improved biosecurity protocols; (*ii*) well-coordinated, large-scale management; (*iii*) targeted research to improve pre- and post-invasion mitigation; and (*iv*) horizon scanning to include a consideration of costs [92]. Well-communicated, timely actions and coordination substantially mitigate the negative impacts of invasive alien species [75, 76, 93, 94]. Based on the projected increases in future costs, we urge the European Union member states to expand their efforts in Union-wide coordination to combat the threat posed by invasive alien species. We further urge investments in a more granular understanding of management-expenditure efficiency that will allow for a more comprehensive idea of the costs of management actions that contribute to lowering invasion costs. This can in turn be expected to help capture with more accuracy the true toll of biological invasions, and identify and fill any knowledge gaps.

Cost assessments play an important role in improving management efficiency and effectiveness by promoting early, focused, and evidence-based management interventions towards alien species, ultimately leading to long-term cost savings, relieving the burden on taxpayers, and enabling governments to allocate their financial resources more effectively. This is further substantiated because prior work has emphasised the ability of early investments (e.g., in biosecurity) to lower impacts of biological invasions in the long term [75, 93]. However, economic downplaying of risks or impacts, often triggered by insufficient budget allocation for conservation, further reduces the likelihood of cooperation among and within countries to implement risk assessments and management planning. Additional economic burdens imposed by invasive alien species could exacerbate an already difficult period of geopolitical tension [95] regarding the cost of natural resources and biodiversity. The identified growing socio-economic impacts of invasive alien species [9, 80] and the current persistent misallocation of strategic investment [96], mean that invasive alien species are degrading many aspects of human life, and placing an additional burden on human and social well-being [2]. Rising resource losses and damages due to invasive alien species could fuel further political debates among European Union member states and affect conservation and research budgets. This relative instability could weaken the European Union in the future following recent crises (e.g., COVID-19 pandemic, Brexit, Russian invasion of Ukraine) [97–99] and could drive policy changes that have negative consequences for trade and biosecurity. For example, the case of changing food-export pathways and cost increases in selling prices received by domestic producers for their output occurred after the invasion of Ukraine [100], a phenomenon that could in turn reduce attention on environmental challenges such as biological invasions. Considering these ongoing economic stressors within the European Union [98], our results warn against the pattern of reduced investment in environmental management [101, 102]. Without improved actions, the rates of biological invasions and associated costs will continue to rise, degrading natural capital, and hampering industries, sustainable development, and sustainability targets [103].

## Supplementary Information

**Additional file 1**: Description of the sectors considered in the InvaCost database. **Additional file 2**: Site-level costs recorded in InvaCost for the European Union member states (a), by type of cost (b) and impacted sector (c) with and without high-leverage points and the respective projected additional cost and percent increase. **Additional file 3**: Detailed breakdown of the considered models included in the model averaging. **Table 1**: Country-level analyses. Values of the fitted parameters for each predictor combination and model performance estimated using Akaike’s information criterion (AIC). We calculated Akaike’s weights for model averaging following Burnham & Anderson [57]. **Table 2**: Country-level analyses without high-leverage points. Values of the fitted parameters for each predictor combination and model performance estimated using Akaike’s information criterion (AIC). Model outputs for each combination of predictors. We calculated Akaike’s weights for model averaging following Burnham & Anderson [57]. **Table 3**: Site-level analyses (A) with all data and (B) without high-leverage points. Values of the fitted parameters for each predictor combination and model performance estimated using Akaike’s information criterion (AIC). Model outputs for each combination of predictors. We calculated Akaike’s weights for model averaging following Burnham & Anderson [57]. **Additional file 4**: Invasion costs (total costs and highly reliable, observed costs) for European Union member states recorded in *InvaCost* v4.1, in 2017 US$ billion. **Additional file 5**: Comparison of the number of established alien species in the European Union and alien species in *InvaCost* v4.1. **Additional file 6**. Established alien species of Union concern recorded among the established alien species in the European Union with the first European Union member state they were recorded in and the year of first record [48]. **Additional file 7**: Breakdown of interpolated costs excluding high-leverage points. **Additional file 8**: Country-level costs recorded in *InvaCost* with and without high-leverage points and the respective projected additional cost and percent increase. **Additional file 9**: Country-level costs recorded in *InvaCost* by type of cost (a) and impacted sector (b) with and without extreme values and the respective projected additional cost and percent increase. **Additional file 10**: Temporal projection of total annual management costs (a), recorded species per year with management costs (b), and number of reported references on management expenditure in *InvaCost* v4.1 per year (c) using linear (left, in blue) and quadratic (right, in orange) robust regressions and their respective confidence intervals. Solid dots and lines represent trends without the inclusion of extreme values. Open circles and dashed lines represent the respective trends when including all data (i.e., including extreme values). Grey dots present data for the period 1960–1979 that are not considered in the projection due to an absence of repeated measures of individual cost entries.

## Data Availability

All data generated or analysed during this study are openly available: github.com/LeungEcoLab/EU_costs.
