# Supplementary material for: Unveiling the hidden economic toll of biological invasions in the European Union
Source: Environ Sci Eur. 2023 Jun 8;35(1):43. doi: 10.1186/s12302-023-00750-3 (PMC10249565; doi:10.1186/s12302-023-00750-3)
Supplement: Supplementary file 1 — Additional file 1: Description of the sectors considered in the InvaCost database. Additional file 2: Site-level costs recorded in InvaCost for the European Union member states (a), by type of cost (b) and impacted sector (c) with and without high-leverage points and the respective projected additional cost and percent increase. Additional file 3: Detailed breakdown of the considered models included in the model averaging. Table 1: Country-level analyses. Values of the fitted parameters for each predictor combination and model performance estimated using Akaike’s information criterion (AIC). We calculated Akaike’s weights for model averaging following Burnham & Anderson [57]. Table 2: Country-level analyses without high-leverage points. Values of the fitted parameters for each predictor combination and model performance estimated using Akaike’s information criterion (AIC). Model outputs for each combination of predictors. We calculated Akaike’s weights for model averaging following Burnham & Anderson [57]. Table 3: Site-level analyses (A) with all data and (B) without high-leverage points. Values of the fitted parameters for each predictor combination and model performance estimated using Akaike’s information criterion (AIC). Model outputs for each combination of predictors. We calculated Akaike’s weights for model averaging following Burnham & Anderson [57]. Additional file 4: Invasion costs (total costs and highly reliable, observed costs) for European Union member states recorded in InvaCost v4.1, in 2017 US$ billion. Additional file 5: Comparison of the number of established alien species in the European Union and alien species in InvaCost v4.1. Additional file 6. Established alien species of Union concern recorded among the established alien species in the European Union with the first European Union member state they were recorded in and the year of first record [48]. Additional file 7: Breakdown of interpolated costs excluding high-leverage points. Additional [file 12302_2023_750_MOESM1_ESM.docx]

**Supplementary Information**

**Additional file 1:** Description of the sectors considered in the *InvaCost* database

| **Sector** | **Description** |
| --- | --- |
| **Agriculture** | In its broadest sense, food and other useful products produced by human activities through using natural and/plant resources from their ecosystems (e.g., crop growing, livestock breeding, beekeeping, land management) |
| **Authorities-stakeholders** | Government services and/or official organisations (e.g., conservation agencies, forest services, associations) that allocate efforts for the management *sensu lato* of biological invasions (e.g., control programs, eradication campaigns, research funding) |
| **Environment** | Impacts on natural resources, ecological processes and/or ecosystem services that have been valued by authors such as disruption of native habitats or degradation of local habitats |
| **Fisheries** | Fish-based activities and services such as fishing and aquaculture |
| **Forestry** | Forest-based activities and services such as timber production/industries and private forests |
| **Health** | Every item directly or indirectly related to the health status of people such as vector control, medical care, and other derived damage on human productivity and well-being |
| **Public and social welfare** | Activities, goods, or services contributing — directly or indirectly — to human well-being and safety, including local infrastructure (e.g., electric system), quality of life (e.g., income, recreation), personal goods (e.g., private property, lands), public services (e.g., transport, water regulation), and market activities (e.g., tourism, trade) |

**Additional file 2:** Site-level costs recorded in *InvaCost* for the European Union member states (a), by type of cost (b) and impacted sector (c) with and without high-leverage points and the respective projected additional cost and percent increase.

| **(a) European Union member state** | **InvaCost v4.1 site-level costs in (US$**  **million)** | **InvaCost v4.1 site-level costs (no high-leverage points, US$ million)** | **Projected additional costs (US$ million)** | **Projected additional costs (no high-leverage points, US$ million)** | **Increase in projected costs (in %)** | **Increase in projected costs (no high-leverage points, in %)** |
| --- | --- | --- | --- | --- | --- | --- |
| Austria | NA | NA | 20.97 | 10.29 | NA | NA |
| Belgium | 0.71 | 0.71 | 34.15 | 19.91 | 4791.76 | 2,794.13 |
| Bulgaria | NA | NA | 23.19 | 13.92 | NA | NA |
| Croatia | NA | NA | 33.04 | 15.54 | NA | NA |
| Cyprus | NA | NA | 6.89 | 3.65 | NA | NA |
| Czech Rep. | NA | NA | 14.49 | 7.99 | NA | NA |
| Denmark | 0.15 | 0.15 | 13.04 | 5.80 | 8,568.86 | 3,809.13 |
| Estonia | 0.11 | 0.11 | 0.49 | 0.26 | 443.75 | 234.51 |
| Finland | 0.75 | 0.75 | 5.93 | 2.16 | 795.99 | 290.06 |
| France | 241.93 | 106.65 | 83.56 | 52.79 | 34.54 | 49.50 |
| Germany | 0.42 | 0.42 | 58.34 | 34.98 | 13,898.07 | 8,332.46 |
| Greece | 27.87 | 27.87 | 23.71 | 10.16 | 85.06 | 36.44 |
| Hungary | NA | NA | 15.20 | 5.12 | NA | NA |
| Ireland | 0.01 | 0.01 | 7.85 | 3.54 | 98,760.67 | 44,521.90 |
| Italy | 90.85 | 90.85 | 14.82 | 10.42 | 16.31 | 11.47 |
| Latvia | NA | NA | 1.66 | 1.00 | NA | NA |
| Lithuania | NA | NA | 1.50 | 0.97 | NA | NA |
| Luxembourg | NA | NA | 12.30 | 5.36 | NA | NA |
| Malta | NA | NA | 20.97 | 10.77 | NA | NA |
| Netherlands | 0.59 | 0.59 | 47.64 | 26.33 | 8,136.62 | 4,498.29 |
| Poland | NA | NA | 9.71 | 5.63 | NA | NA |
| Portugal | 36.13 | 0.56 | 33.17 | 17.34 | 91.80 | 3102.04 |
| Romania | NA | NA | 26.79 | 17.00 | NA | NA |
| Slovakia | NA | NA | 29.54 | 16.57 | NA | NA |
| Slovenia | NA | NA | 33.14 | 15.91 | NA | NA |
| Spain | 22.53 | 22.53 | 108.31 | 19.27 | 480.76 | 85.53 |
| Sweden | NA | NA | 21.92 | 10.75 | NA | NA |
| Average | 35.17 | 20.93 | 26.01 | 12.72 | 11,342.02 | 5647.12 |
| Total Site | 422.04 | 251.19 | 702.31 | 343.45 | 166.41 | 136.73 |
|  |  |  |  |  |  |  |
| **(b) Type of cost** | **InvaCost v4.1 site-level costs in (US$**  **million)** | **InvaCost v4.1 site-level costs (no high-leverage points, US$ million)** | **Projected additional costs (US$ million)** | **Projected additional costs (no high-leverage points, US$ million)** | **Increase in projected costs (in %)** | **Increase in projected costs (no high-leverage points, in %)** |
| Damage | 294.27 | 123.42 | 263.26 | 94.17 | 89.46 | 76.30 |
| Management | 117.90 | 117.90 | 310.85 | 189.34 | 263.65 | 160.59 |
| Mixed | 9.87 | 9.87 | 128.20 | 59.95 | 1,299.40 | 607.58 |
| Average | 140.68 | 83.73 | 234.10 | 114.48 | 550.84 | 281.49 |
| Total Site | 422.04 | 251.19 | 702.31 | 343.45 | 166.41 | 136.73 |
| (**c) Impacted sector** | **InvaCost v4.1 site-level costs in (US$**  **million)** | **InvaCost v4.1 site-level costs (no high-leverage points, US$ million)** | **Projected additional costs (US$ million)** | **Projected additional costs (no high-leverage points, US$ million)** | **Increase in projected costs (in %)** | **Increase in projected costs (no high-leverage points, in %)** |
| Agriculture | 52.59 | 17.01 | 176.40 | 53.42 | 335.45 | 313.96 |
| Authorities-Stakeholders | 70.67 | 70.67 | 110.39 | 72.55 | 156.20 | 102.66 |
| Diverse | 65.78 | 65.78 | 217.09 | 131.86 | 330.04 | 200.47 |
| Fishery | 0.40 | 0.40 | 2.62 | 1.26 | 659.42 | 317.16 |
| Forestry | 5.02 | 5.02 | 58.82 | 26.10 | 1,170.93 | 519.62 |
| Health | 224.16 | 88.88 | 99.74 | 36.27 | 44.49 | 40.81 |
| Public and social welfare | 3.42 | 3.42 | 37.24 | 21.98 | 1,088.84 | 642.67 |
| Average | 60.29 | 35.88 | 100.33 | 49.06 | 540.77 | 305.34 |
| Total Site | 422.04 | 251.19 | 702.31 | 343.45 | 166.41 | 136.73 |

**Additional file 3:** Detailed breakdown of the considered models included in the model averaging.

| **Table 1:** Country-level analyses. Values of the fitted parameters for each predictor combination and model performance estimated using Akaike’s information criterion (AIC). We calculated Akaike’s weights for model averaging following Burnham & Anderson [11]. | | | | | | | | | |
| --- | --- | --- | --- | --- | --- | --- | --- | --- | --- |
| **Model** | **Predictors** | **Fitted parameter values ± uncertainty** | | | | **AIC** | **ΔAIC** | **Estimated cost before model averaging (billion $)** | **Akaike’s weights** |
| m0 | None |  |  |  |  | 1339.35 | 64.18 | 8.27 | 0 |
| m1 | GDP | -0.06 (-0.21, 0.08) |  |  |  | 1340.89 | 65.72 | 9.16 | 0 |
| m2 | Population size | 0.07 (-0.08, 0.23) |  |  |  | 1340.95 | 65.78 | 7.6 | 0 |
| m3 | Country Area | 0.17 (-0.04, 0.37) |  |  |  | 1339.64 | 64.47 | 6.89 | 0 |
| m4 | Sector | 1.88 (0.59, 2.35) |  |  |  | 1299.5 | 24.33 | 8.06 | 0 |
| m5 | GDP + population size | -1.59 (-2.18, -0.75) | 1.95 (0.79, 2.85) |  |  | 1314.82 | 39.65 | 14.69 | 0 |
| m6 | GDP + sector | 0.07 (-0.08, 0.18) | 2 (0.66, 2.48) |  |  | 1300.15 | 24.98 | 7.15 | 0 |
| m7 | GDP + area | -0.75 (-1.07, -0.23) | 1.1 (0.28, 1.53) |  |  | 1323.42 | 48.24 | 17.14 | 0 |
| m8 | Population size + sector | 0.22 (0, 0.36) | 2.05 (0.68, 2.43) |  |  | 1294.75 | 19.58 | 6.37 | 0 |
| m9 | Area + sector | 0.2 (-0.07, 0.41) | 1.93 (0.63, 2.35) |  |  | 1297.36 | 22.18 | 6.51 | 0 |
| m10 | Population size + area | -0.45 (-0.69, 0.12) | 0.65 (-0.08, 0.97) |  |  | 1338.89 | 63.71 | 9 | 0 |
| m11 | GDP + area + sector | -0.18 (-0.53, 0.21) | 0.43 (-0.23, 0.93) | 1.71 (0.56, 2.38) |  | 1297.72 | 22.55 | 7.23 | 0 |
| m12 | GDP + population size + area | -1.49 (-2.16, -0.91) | 1.62 (0.7, 3.17) | 0.28 (-0.81, 0.91) |  | 1316.51 | 41.33 | 12.91 | 0 |
| m13 | GDP + population size + sector | -0.73 (-1.45, -0.12) | 1.12 (0.26, 2.05) | 1.57 (0.31, 2.16) |  | 1281.1 | 5.93 | 8.08 | 0.05 |
| m14 | Population size + area + sector | 0.42 (0.09, 0.87) | -0.26 (-0.89, 0.22) | 2.15 (1.01, 2.6) |  | 1295.81 | 20.64 | 7.43 | 0 |
| m15 | GDP + population size + area + sector | -0.9 (-1.63, -0.28) | 2.05 (0.75, 3.05) | -0.93 (-1.39, 0.02) | 1.81 (0.6, 2.34) | 1275.17 | 0 | 24.2 | 0.95 |
| **Table 2:** country-level analyses without high-leverage points. Values of the fitted parameters for each predictor combination and model performance estimated using Akaike’s information criterion (AIC). Model outputs for each combination of predictors. We calculated Akaike’s weights for model averaging following Burnham & Anderson [11]. | | | | | | | | | |
| **Model** | **Predictors** | **Fitted parameter values ± uncertainty** | | | | **AIC** | **ΔAIC** | **Estimated cost before model averaging (billion $)** | **Akaike’s weights** |
| m0 | None |  |  |  |  | 1138.38 | 1.17 | 7.88 | 0.08 |
| m1 | GDP | 0.12 (-0.16, 0.3) |  |  |  | 1138.75 | 1.54 | 6.63 | 0.07 |
| m2 | Population size | 0.19 (-0.05, 0.39) |  |  |  | 1137.21 | 0 | 6.46 | 0.15 |
| m3 | Country Area | 0.18 (-0.05, 0.38) |  |  |  | 1137.93 | 0.73 | 6.5 | 0.11 |
| m4 | Sector | -0.07 (-0.76, 0.43) |  |  |  | 1140.32 | 3.12 | 7.9 | 0.03 |
| m5 | GDP + population size | -0.48 (-1.21, 0.13) | 0.7 (0.11, 1.52) |  |  | 1137.26 | 0.05 | 7.71 | 0.15 |
| m6 | GDP + sector | 0.13 (-0.16, 0.32) | 0.06 (-0.7, 0.58) |  |  | 1140.7 | 3.49 | 6.54 | 0.03 |
| m7 | GDP + country area | -0.03 (-0.5, 0.33) | 0.21 (-0.17, 0.61) |  |  | 1139.91 | 2.71 | 6.6 | 0.04 |
| m8 | Population size + sector | 0.2 (-0.05, 0.41) | 0.05 (-0.83, 0.57) |  |  | 1139.16 | 1.96 | 6.41 | 0.06 |
| m9 | Area + sector | 0.18 (-0.06, 0.39) | -0.08 (-0.89, 0.49) |  |  | 1139.84 | 2.64 | 6.51 | 0.04 |
| m10 | Population size + area | 0.21 (-0.23, 0.73) | -0.02 (-0.56, 0.35) |  |  | 1139.2 | 1.99 | 6.51 | 0.06 |
| m11 | GDP + country area + sector | -0.25 (-0.56, 0.35) | 0.42 (-0.14, 0.65) | -0.36 (-0.98, 0.35) |  | 1141.39 | 4.19 | 8.1 | 0.02 |
| m12 | GDP + population size + area | -0.5 (-1.32, 0.08) | 0.81 (0.22, 2.01) | -0.1 (-0.75, 0.26) |  | 1139.19 | 1.98 | 8.05 | 0.06 |
| m13 | GDP + population size + sector | -0.66 (-1.24, 0.06) | 0.85 (0.2, 1.54) | -0.27 (-1.1, 0.35) |  | 1138.77 | 1.57 | 8.68 | 0.07 |
| m14 | Population size + country area + sector | 0.27 (-0.2, 0.79) | -0.09 (-0.62, 0.32) | 0.1 (-0.81, 0.64) |  | 1141.1 | 3.89 | 6.61 | 0.02 |
| m15 | GDP + population size + country area + sector | -0.67 (-1.3, 0.02) | 0.73 (0.24, 2.02) | 0.14 (-0.77, 0.35) | -0.36 (-1.05, 0.45) | 1140.68 | 3.48 | 8.71 | 0.03 |

**Table 3:** Site-level analyses (A) with all data and (B) without high-leverage points. Values of the fitted parameters for each predictor combination and model performance estimated using Akaike’s information criterion (AIC). Model outputs for each combination of predictors. We calculated Akaike’s weights for model averaging following Burnham & Anderson [11].

| **Model** | **Predictors** | **Fitted parameter values ± uncertainty** | | | **AIC** | **ΔAIC** | **Estimated cost before model averaging (billion $)** | **Akaike’s weights** |
| --- | --- | --- | --- | --- | --- | --- | --- | --- |
| **A) All data** | | | | | | | | |
| m0 | None |  |  |  | 12015.85 | 0 | 0.51 | 0.36 |
| m1 | GDP | 0.11 (-0.95, 0.37) |  |  | 12015.89 | 0.04 | 0.44 | 0.35 |
| m2 | Population size | 0.23 (-1.19, 0.49) |  |  | 12016.43 | 0.58 | 0.41 | 0.27 |
| m3 | Sector | -0.67 (-0.8, 1.95) |  |  | 12017.82 | 1.97 | 0.49 | 0.13 |
| m4 | GDP + population size | 0.04 (-1.36, 0.42) | 0.1 (-0.58, 1.5) |  | 12017.87 | 2.02 | 0.44 | 0.13 |
| m5 | GDP + sector | 0.11 (-0.99, 0.34) | 0.18 (-0.44, 1.48) |  | 12017.89 | 2.04 | 0.45 | 0.13 |
| m6 | Sector + pop size | 0.15 (-1.19, 0.53) | 0.17 (-0.14, 1.47) |  | 12017.87 | 2.02 | 0.44 | 0.13 |
| m7 | GDP + population size + sector | -0.1 (-1.43, 0.33) | 0.28 (-0.53, 1.58) | 0.22 (-0.71, 2.11) | 12019.87 | 4.02 | 0.43 | 0.05 |
| **B) No high-leverage points** | | | | | | | | |
| m0 | None |  |  |  | 4795.43 | 37.01 | 0.45 | 0 |
| m1 | GDP | 0.11 (-1.03, 0.29) |  |  | 4765.16 | 6.74 | 0.39 | 0.01 |
| m2 | Population size | 0.15 (-1.25, 0.38) |  |  | 4764.71 | 6.29 | 0.37 | 0.02 |
| m3 | Sector | -4.42 (-5.68, 1.22) |  |  | 4789.15 | 30.72 | 0.41 | 0 |
| m4 | GDP + population size | -0.34 (-1.93, 0.64) | 0.6 (-0.96, 2.22) |  | 4766.16 | 7.73 | 0.35 | 0.01 |
| m5 | GDP + sector | 0.11 (-1.03, 0.29) | -4.44 (-5.58, 0.74) |  | 4758.99 | 0.56 | 0.35 | 0.31 |
| m6 | Sector + population size | 0.15 (-1.25, 0.38) | -4.44 (-5.56, 0.74) |  | 4758.42 | 0 | 0.34 | 0.42 |
| m7 | GDP + population size + sector | -0.35 (-1.92, 0.66) | 0.61 (-1.04, 2.07) | -4.3 (-5.25, 0.95) | 4759.61 | 1.18 | 0.32 | 0.23 |

**Additional file 4:** Invasion costs (total costs and highly reliable, observed costs) for European Union member states recorded in *InvaCost* v4.1, in 2017 US$ billion.

| **Country** | **Total costs** | **highly reliable, observed costs** |
| --- | --- | --- |
| Austria | 1.72 | 0.13 |
| Belgium | 1.53 | 0.01 |
| Bulgaria | 1.53 | 0.31 |
| Croatia | 1.02 | 0.19 |
| Cyprus | 0.12 | 0 |
| Czech Rep. | 5.89 | 0.03 |
| Denmark | 2.17 | 0 |
| Estonia | 0.11 | 0 |
| Finland | 1.13 | 0.06 |
| France | 26.26 | 0.88 |
| Germany | 16.85 | 0.79 |
| Greece | 4.81 | 0.05 |
| Hungary | 3.16 | 0.92 |
| Ireland | 0.61 | 0.28 |
| Italy | 3.43 | 0.52 |
| Latvia | 0.2 | 0 |
| Lithuania | 0.43 | 0 |
| Luxembourg | 0.07 | 0 |
| Malta | 0.02 | 0 |
| Netherlands | 5.65 | 0.26 |
| Poland | 2.71 | 0.02 |
| Portugal | 20.46 | 0.75 |
| Romania | 5.09 | 1.17 |
| Slovakia | 0.78 | 0.15 |
| Slovenia | 0.25 | 0.03 |
| Spain | 22.45 | 0.4 |
| Sweden | 1.39 | 0.39 |
| *TOTAL* | *129.9* | *7.34* |

**Additional file 5:** Comparison of the number of established alien species in the European Union and alien species in *InvaCost* v4.1.

| **Country** | **Established alien species in Europe** | **Species of Union Concern**  **(and those with records in InvaCost)** | **Invasive alien species in InvaCost** | **Invasive alien species with highly reliable, observed costs in InvaCost** | **Invasive alien species with highly reliable, observed country-level costs in InvaCost** | **Established alien species of Union concern (2016; n = 48)** | **Established alien species of Union concern in Europe (2019; n = 66)** | **Species of Union concern recorded in InvaCost (2019; based on n = 66)** | **Total entries in InvaCost based on the 66 species of union concern** | **Highly reliable, observed entries in InvaCost based on the 66 species of union concern** | **Highly reliable, observed country-level entries in InvaCost based on the 66 species of union concern** |
| --- | --- | --- | --- | --- | --- | --- | --- | --- | --- | --- | --- |
| Austria | 1553 | 20 (0) | 3 | 2 | 2 | 18 | 20 | 0 | 0 | 0 | 0 |
| Belgium | 2678 | 37 (5) | 8 | 5 | 0 | 33 | 36 | 5 | 19 | 18 | 0 |
| Bulgaria | 1077 | 116 (0) | 2 | 1 | 1 | 8 | 11 | 0 | 0 | 0 | 0 |
| Croatia | 924 | 15 (0) | 2 | 1 | 1 | 11 | 15 | 0 | 0 | 0 | 0 |
| Cyprus | 611 | 4 (0) | 4 | 2 | 2 | 3 | 4 | 1 | 0 | 0 | 11 |
| Czech Rep. | 1770 | 14 (1) | 2 | 2 | 2 | 9 | 13 | 8 | 40 | 19 | 5 |
| Denmark | 2693 | 19 (8) | 23 | 4 | 3 | 18 | 19 | 0 | 0 | 0 | 0 |
| Estonia | 939 | 10 (0) | 1 | 1 | 0 | 8 | 9 | 4 | 32 | 32 | 32 |
| Finland | 360 | 10 (4) | 15 | 4 | 2 | 10 | 10 | 26 | 336 | 334 | 1 |
| France | 3691 | 48 (26) | 67 | 63 | 5 | 38 | 47 | 4 | 80 | 78 | 72 |
| Germany | 2419 | 29 (4) | 21 | 13 | 11 | 26 | 29 | 0 | 0 | 0 | 0 |
| Greece | 909 | 29 (0) | 4 | 2 | 1 | 9 | 13 | 0 | 0 | 0 | 0 |
| Hungary | 412 | 26 (0) | 2 | 1 | 1 | 23 | 26 | 2 | 10 | 10 | 0 |
| Ireland | 1713 | 20 (2) | 2 | 12 | 5 | 20 | 20 | 4 | 50 | 49 | 23 |
| Italy | 3056 | 46 (4) | 17 | 12 | 5 | 35 | 46 | 0 | 0 | 0 | 0 |
| Latvia | 730 | 11 (0) | 0 | 0 | 0 | 10 | 11 | 1 | 1 | 1 | 1 |
| Lithuania | 499 | 9 (1) | 1 | 1 | 1 | 7 | 8 | 0 | 0 | 0 | 0 |
| Luxembourg | 217 | 11 (0) | 1 | 1 | 0 | 10 | 11 | 0 | 0 | 0 | 0 |
| Malta | 472 | 7 (0) | 1 | 1 | 1 | 6 | 7 | 27 | 133 | 131 | 128 |
| Netherlands | 3049 | 33 (27) | 37 | 10 | 5 | 30 | 33 | 0 | 0 | 0 | 0 |
| Poland | 1598 | 21 (0) | 2 | 1 | 1 | 17 | 21 | 2 | 14 | 5 | 1 |
| Portugal | 1208 | 25 (2) | 10 | 5 | 3 | 17 | 24 | 0 | 0 | 0 | 0 |
| Romania | 633 | 16 (0) | 2 | 1 | 1 | 13 | 16 | 0 | 0 | 0 | 0 |
| Slovakia | 612 | 14 (0) | 2 | 1 | 1 | 12 | 14 | 0 | 0 | 0 | 0 |
| Slovenia | 924 | 16 (0) | 2 | 1 | 1 | 13 | 16 | 18 | 309 | 294 | 22 |
| Spain | 1859 | 37 (18) | 161 | 158 | 19 | 26 | 34 | 6 | 8 | 7 | 5 |
| Sweden | 2213 | 37 (6) | 23 | 13 | 13 | 28 | 32 | 0 | 0 | 0 | 0 |
| Mean | 1437.7 | 20.8 (4) | 15.4 | 11.8 | 3.3 | 17.0 | 20.2 | 3.9 | 47.7 | 45.5 | 12.4 |
| Europe | 13331 | 66 (48) | 259 | 221 | 49 | 48 | 66 | 48 | 1288 | 1228 | 336 |

**Additional file 6.** Established alien species of Union concern recorded among the established alien species in the European Union with the first European Union member state they were recorded in and the year of first record [86].

| **Species of Union Concern with costs reported in InvaCost** | **EU member state of first record** | **Year of first record** |
| --- | --- | --- |
| *Acacia saligna* | NA | NA |
| *Baccharis halimifolia* | France | 1683 |
| *Cabomba caroliniana* | NA | NA |
| *Callosciurus erythraeus* | France | 1974 |
| *Corvus splendens* | Netherlands | 1994 |
| *Eichhornia crassipes* | Portugal | 1936 |
| *Elodea nuttallii* | France | 1973 |
| *Eriocheir sinensis* | Germany | 1912 |
| *Fundulus heteroclitus* | Spain | 1973 |
| *Hakea sericea* | NA | NA |
| *Heracleum mantegazzianum* | Sweden | 1600 |
| *Heracleum persicum* | NA | NA |
| *Heracleum sosnowskyi* | NA | NA |
| *Herpestes javanicus* | NA | NA |
| *Humulus scandens* | NA | NA |
| *Hydrocotyle ranunculoides* | Belgium | 1992 |
| *Impatiens glandulifera* | France | 1839 |
| *Lagarosiphon major* | France | 1939 |
| *Lepomis gibbosus* | Spain | 1910 |
| *Lithobates catesbeianus* | France | 1804 |
| *Ludwigia grandiflora* | France | 1823 |
| *Ludwigia peploides* | France | 1827 |
| *Lysichiton americanus* | NA | NA |
| *Muntiacus reevesi* | Netherlands | 1998 |
| *Myocastor coypus* | France | 1929 |
| *Myriophyllum aquaticum* | France | 1880 |
| *Nasua nasua* | NA | NA |
| *Nyctereutes procyonoides* | Denmark | 1930 |
| *Ondatra zibethicus* | Germany | 1914 |
| *Pacifastacus leniusculus* | Finland | 1931 |
| *Parthenium hysterophorus* | NA | NA |
| *Perccottus glenii* | NA | NA |
| *Persicaria perfoliata* | NA | NA |
| *Pistia stratiotes* | NA | NA |
| *Procambarus clarkii* | Spain | 1973 |
| *Procyon lotor* | France | 1934 |
| *Pseudorasbora parva* | Netherlands | 1998 |
| *Pueraria montana* | NA | NA |
| *Pycnonotus cafer* | NA | NA |
| *Salvinia molesta* | NA | NA |
| *Sciurus carolinensis* | Ireland | 1911 |
| *Sciurus niger* | NA | NA |
| *Solenopsis invicta* | NA | NA |
| *Tamias sibiricus* | Netherlands | 1972 |
| *Threskiornis aethiopicus* | France | 1975 |
| *Trachemys scripta* | NA | NA |
| *Wasmannia auropunctata* | NA | NA |
| *Xenopus laevis* | France | 1982 |

**Additional file 7:** Breakdown of interpolated costs excluding high-leverage points.

Without reported annualised costs of > $500 million as reported damage costs from Romania and Hungary, the highest increase occurred in Lithuania (973,026%), but was then followed by Finland (36,382%), and Denmark (24,388%). Excluding high-leverage points, France had the highest increase (US$556.1 million; €582.6 million), followed by Romania (US$444.5 million; €465.7 million), Ireland (US$427.6 million; €448.0 million), and Czech Republic (US$415.2 million; €435.0 million). Added costs for all other countries were < US$400 million (€419.0 million). We provide a detailed breakdown of projected country-level costs in Figure 1 (below) and Supplements 9 and 10. On average, projection of costs increased national invasion costs by 63,092% (± 232,195; 48,585% without outliers). We further found that the estimation of costs in European Union member states increased by an average of 2,640% (± 2831%; 751% without high-leverage points values) for impacted sectors (Supplement 10). Following the projection, costs to public and social welfare increased the most (994% without high-leverage points values to 7,950%), followed by costs to forestry (652 to 4,587%), authorities and stakeholders (957 to 2,611%), fishery (1,106 to 2,111%), but with only limited increases for agriculture (18 to 56%) and the health sector (28 to 30%) (see Supplement 1 for a detailed description of the sectors considered in the *InvaCost* database). Projection increased damage costs by 170% without high-leverage points values to 325%, and by 976% to 3,933% for management costs (Supplement 10).

The considered alternative models using all possible combinations of our four macroeconomic variables and calculated Akaike's information criterion (AIC) for each model are displayed in Table 3 of Supplement 4.


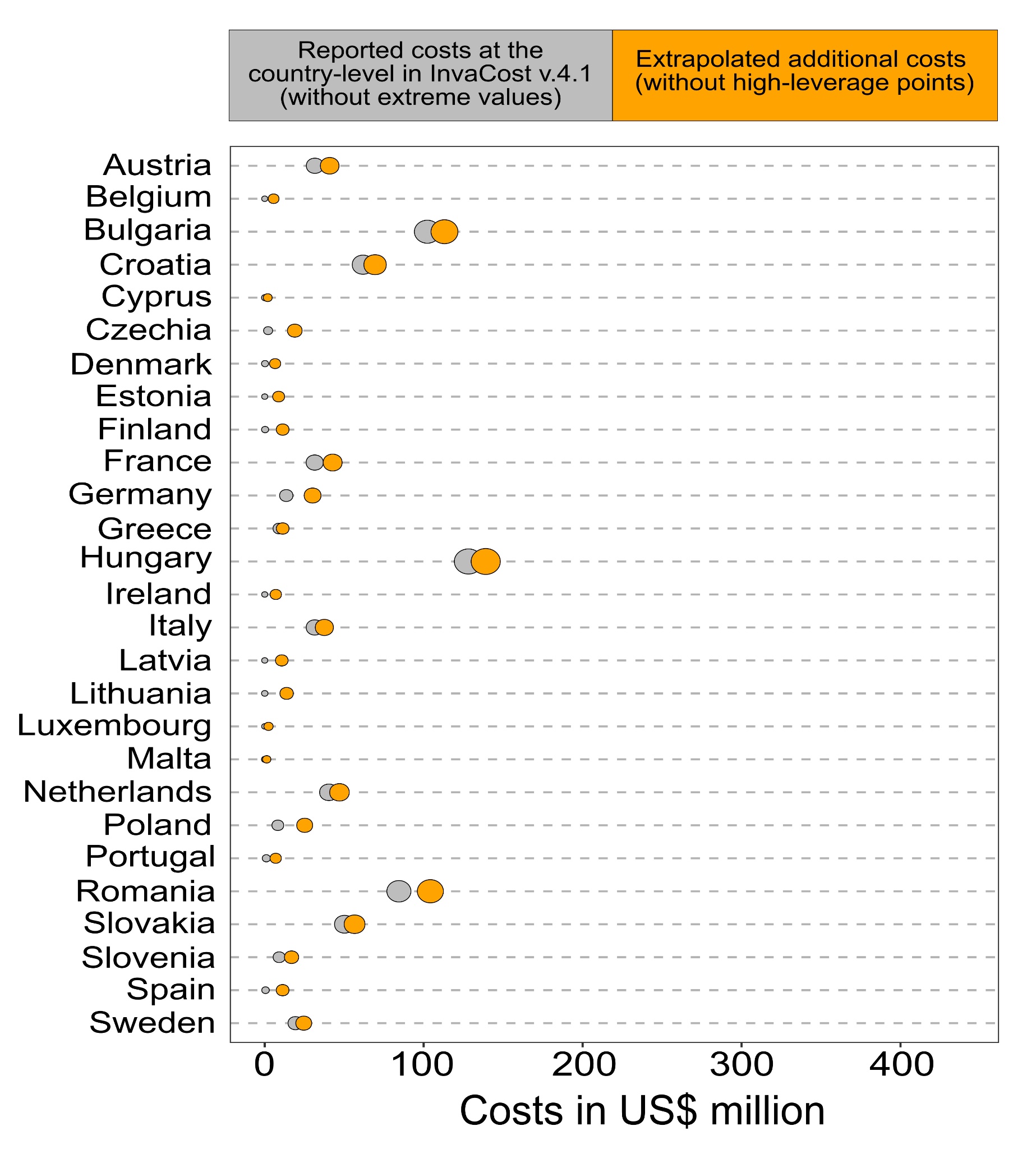


**Figure 1:** Comparison between highly reliable observed country-level costs and projected costs with (**a**) and without high-leverage points at the level of individual European Union member states (**b**). The size of the circles is scaled to the respective cost.

**Additional file 8:** Country-level costs recorded in *InvaCost* with and without high-leverage points and the respective projected additional cost and percent increase.

| **Country** | **InvaCost v4.1 country-level costs in (US$**  **million)** | **InvaCost v4.1 country-level costs (no high-leverage points, US$ million)** | **Projected additional costs (US$ million)** | **Projected additional costs (no high-leverage points, US$ million)** | **Increase in projected costs (in %)** | **Increase in projected costs (no high-leverage points, in %)** |
| --- | --- | --- | --- | --- | --- | --- |
| Austria | 126.29 | 126.29 | 694.05 | 363.35 | 549.59 | 287.71 |
| Belgium | NA | NA | 1,591.82 | 244.68 | NA | NA |
| Bulgaria | 306.86 | 306.86 | 694.62 | 236.80 | 226.37 | 77.17 |
| Croatia | 185.51 | 185.51 | 462.69 | 219.57 | 249.42 | 118.36 |
| Cyprus | 1.14 | 1.14 | 23.83 | 17.87 | 2,089.76 | 1,567.03 |
| Czech. Rep. | 6.35 | 6.35 | 3,818.83 | 415.21 | 60,093.20 | 6,533.81 |
| Denmark | 0.96 | 0.96 | 524.67 | 234.05 | 54,670.40 | 24,388.10 |
| Estonia | NA | NA | 175.46 | 196.77 | NA | NA |
| Finland | 0.72 | 0.72 | 66.80 | 263.64 | 9,218.55 | 36,382.16 |
| France | 377.37 | 377.37 | 2,298.96 | 556.09 | 609.20 | 147.36 |
| Germany | 434.00 | 434.00 | 2,385.48 | 391.73 | 549.65 | 90.26 |
| Greece | 27.00 | 27.00 | 44.75 | 51.97 | 165.74 | 192.49 |
| Hungary | 917.69 | 256.35 | 193.25 | 280.09 | 21.06 | 109.26 |
| Ireland | NA | NA | 264.03 | 427.65 | NA | NA |
| Italy | 346.36 | 346.36 | 1,148.62 | 334.35 | 331.62 | 96.53 |
| Latvia | NA | NA | 252.99 | 236.72 | NA | NA |
| Lithuania | 0.02 | 0.02 | 201.19 | 179.02 | 1,093,524.74 | 973,026.46 |
| Luxembourg | NA | NA | 40.61 | 157.29 | NA | NA |
| Malta | 0.15 | 0.15 | 193.24 | 22.44 | 129,922.73 | 15,086.96 |
| Netherlands | 242.27 | 242.27 | 1,966.52 | 344.42 | 811.72 | 142.17 |
| Poland | 24.72 | 24.72 | 1,962.24 | 379.57 | 7,936.47 | 1,535.22 |
| Portugal | 3.03 | 3.03 | 521.79 | 183.37 | 17,221.70 | 6,052.08 |
| Romania | 1165.38 | 168.82 | 1,345.87 | 444.53 | 115.49 | 263.31 |
| Slovakia | 150.62 | 150.62 | 161.64 | 166.25 | 107.32 | 110.38 |
| Slovenia | 27.30 | 27.30 | 1,802.66 | 283.85 | 6,602.42 | 1,039.64 |
| Spain | 17.94 | 17.94 | 538.28 | 278.62 | 3,000.08 | 1,552.87 |
| Sweden | 308.97 | 308.97 | 29.37 | 260.05 | 9.50 | 84.16 |
|  |  |  |  |  |  |  |
| **Mean** | **212.30** | **136.94** | **866.82** | **265.55** | **63,092.12** | **48,585.61** |
| **Europe** | **4670.67** | **3012.76** | **23,404.27** | **7,169.95** | **501.09** | **237.99** |

**Additional file 9:** Country-level costs recorded in *InvaCost* by type of cost (a) and impacted sector (b) with and without extreme values and the respective projected additional cost and percent increase.

| **(a) Type of cost** | **InvaCost v4.1 country-level costs (million $)** | **InvaCost v4.1 country-level costs (no high-leverage points, million $)** | **Projected additional costs (million $)** | **Projected additional costs (no high-leverage points, million $)** | **Increase in projected costs (in %)** | **Increase in projected costs (no high-leverage points, in %)** |
| --- | --- | --- | --- | --- | --- | --- |
| Damage | 4429.21 | 2771.31 | 14400.38 | 4710.76 | 325.12 | 169.98 |
| Management | 222.90 | 222.90 | 8767.76 | 2175.95 | 3933.43 | 976.19 |
| Mixed | 18.55 | 18.55 | 236.13 | 283.23 | 1272.90 | 1526.80 |
|  |  |  |  |  |  |  |
| **Mean** | **1556.89** | **1004.25** | **7801.42** | **2389.98** | **1843.82** | **890.99** |
| **Europe** | **4670.66** | **3012.76** | **23404.27** | **7169.95** | **501.09** | **237.99** |
|  |  |  |  |  |  |  |
| **(b) Impacted sector** | **InvaCost v4.1 country-level costs (million $)** | **InvaCost v4.1 country-level costs (no high-leverage points, million $)** | **Projected additional costs (million $)** | **Projected additional costs (no high-leverage points, million $)** | **Increase in projected costs (in %)** | **Increase in projected costs (no high-leverage points in %)** |
| Agriculture | 3110.07 | 1452.17 | 549.73 | 810.89 | 17.68 | 55.84 |
| Authorities-stakeholders | 357.50 | 357.50 | 9333.86 | 3420.27 | 2610.86 | 956.71 |
| Diverse | 64.74 | 64.74 | 758.29 | 949.12 | 1171.32 | 1466.09 |
| Fisheries | 4.18 | 4.18 | 88.28 | 46.24 | 2111.55 | 1106.00 |
| Forestry | 177.81 | 177.81 | 8157.39 | 1159.36 | 4587.75 | 652.03 |
| Health | 902.99 | 902.99 | 273.23 | 253.49 | 30.26 | 28.07 |
| Public and social welfare | 53.38 | 53.38 | 4243.49 | 530.58 | 7950.20 | 994.04 |
|  |  |  |  |  |  |  |
| **Mean** | **667.24** | **430.39** | **3343.47** | **1024.28** | **2639.94** | **751.26** |
| **Europe** | **4670.66** | **3012.76** | **23404.27** | **7169.95** | **501.09** | **237.99** |

**Additional file 10:** Temporal projection of total annual management costs (**a**), recorded species per year with management costs (**b**), and number of reported references on management expenditure in *InvaCost* v4.1 per year (**c**) using linear (left, in blue) and quadratic (right, in orange) robust regressions and their respective confidence intervals. Solid dots and lines represent trends without the inclusion of extreme values. Open circles and dashed lines represent the respective trends when including all data (i.e., including extreme values). Grey dots present data for the period 1960–1979 that are not considered in the projection due to an absence of repeated measures of individual cost entries.

**
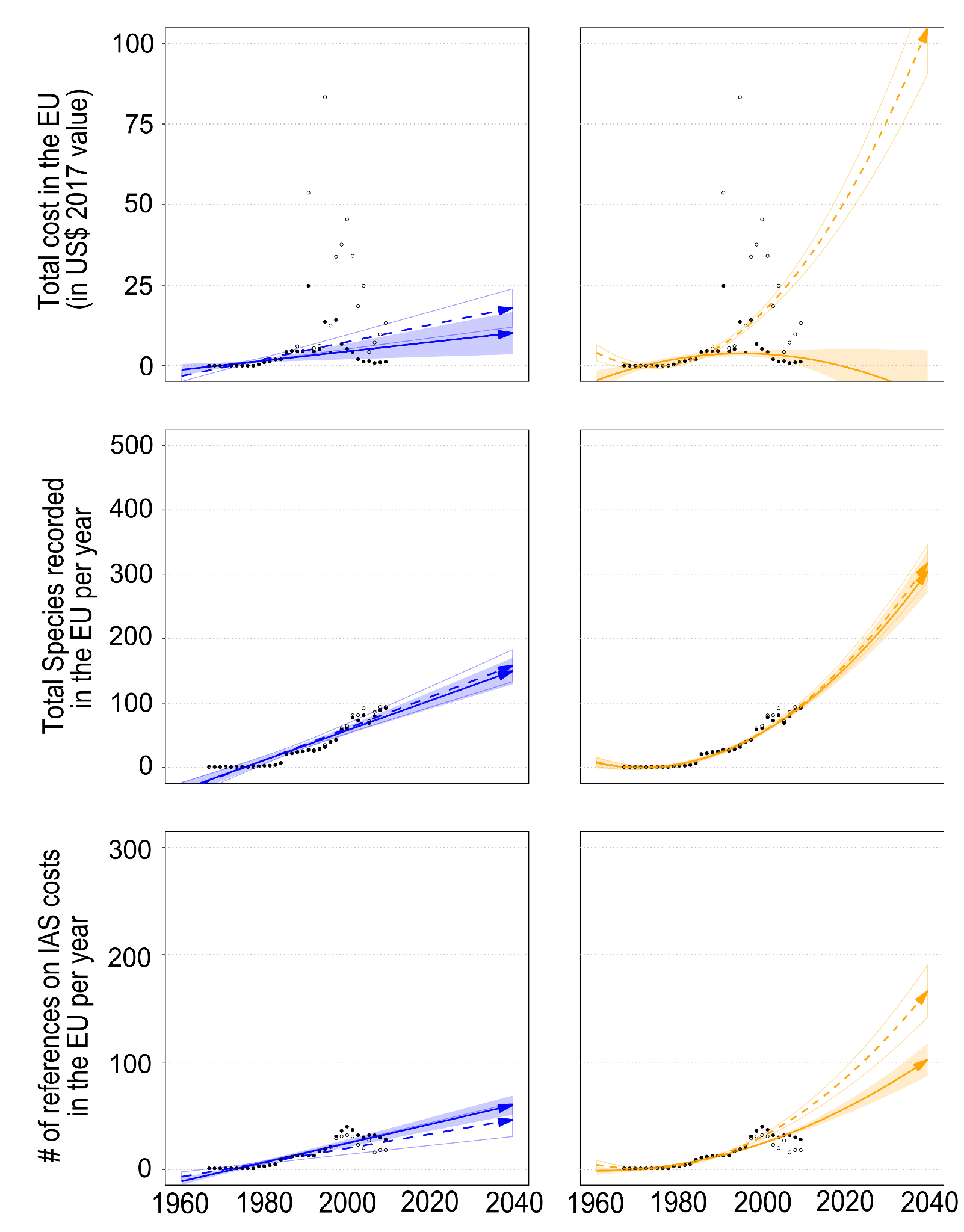
**
